# Kawasaki disease in two sets of monozygotic twins: Is the etiology genetic or environmental?

**DOI:** 10.12669/pjms.291.2713

**Published:** 2013

**Authors:** Xiaomei Zhang, Jinghui Sun, Shubo Zhai, Sirui Yang

**Affiliations:** 1 Xiaomei Zhang, PhD, Department of Pediatrics, The First Hospital of Jilin University, 71 Xinmin Street, Changchun, Jilin 130021, People’s Republic of China.; 2 Jinghui Sun, MD, Department of Pediatrics, The First Hospital of Jilin University, 71 Xinmin Street, Changchun, Jilin 130021, People’s Republic of China.; 3 Shubo Zhai, PhD, Department of Pediatrics, The First Hospital of Jilin University, 71 Xinmin Street, Changchun, Jilin 130021, People’s Republic of China.; 4 Sirui Yang, MD, PhD, Department of Pediatrics, The First Hospital of Jilin University, 71 Xinmin Street, Changchun, Jilin 130021, People’s Republic of China.

**Keywords:** Kawasaki disease, Monozygotic twins, Genetic susceptibility, Infection

## Abstract

Two sets of monozygotic (MZ) twins with Kawasaki disease (KD) from two different families are reported. Twin set 1, previously healthy 71-day-old MZ twin girls were diagnosed with incomplete KD and pneumonia. The symptoms occurred at the same time in both girls. Both girls had ectasia of right coronary arteries. In twin set 2, the younger of 18-month-old MZ twin boys was diagnosed with KD and bronchitis. After 53 days, his elder brother was diagnosed with the same disease. The symptoms occurred at different time, but were almost identical. Neither boy displayed coronary artery changes. These findings support the hypothesis that genes susceptible to KD and coronary-artery lesions may exist in families. The different clinical characteristics among MZ twins from different families also suggest diverse and complex nature of KD.

## Introduction

 The first case series of Kawasaki Disease (KD) was reported in 1967. Kawasaki disease is the most common cause of multisystem vasculitis in children. The coronary arteries are most commonly damaged in KD. The diagnosis is made by presentation of fever for at least 5 days and at least 4 of the following signs: (1) Bilateral conjunctival congestion, (2) Changes of lips and oral cavity: red lips, strawberry tongue, diffuse injection of oral and pharyngeal mucosa, (3) Polymorphous exanthema, (4) Changes of peripheral extremities, (acute phase: Red palms and soles, indurative edema), (convalescent phase: Membranous desquamation from fingertips), (5) Acute nonpurulent cervical lymphadenopathy.

 However, patients with four of the principal criteria can be diagnosed as KD when coronary aneurysm or dilatation is recognized by two-dimensional (2D) echocardiography or coronary angiography.^1^ Although the exact etiology of KD remains unknown, infection and genetic factors are presumed to play consistent role. KD has become an important research topic in the field of pediatric cardiology. Monozygotic (MZ) twins affected with KD may serve as optimum samples to study the disease characteristics as they share a nearly identical genetic code and are likely to be living in similar environments. The reports on KD affecting MZ twins are rare. The PUBMED search revealed only three literatures for the keywords “Kawasaki disease and monozygotic twins.^2^^-^^4^ In this report, two sets of monozygotic (MZ) twins with Kawasaki disease (KD) from two different families are reported, both of them have explicit evidence of respiratory tract infection, especially, twin set 1 were only 71-day-old. Combined effects of infection and hereditary susceptibility seemed to be the etiology for KD.

## Case Reports

***Twin set 1:*** Previously healthy, 71-day-old MZ twin girls presented with fever and cough of 10 days duration and, indurative edema in the hands and feet of three days duration. The symptoms occurred at the same time in both of them. The details of clinical manifestations, part of the laboratory tests and echocardiography findings are shown in Table-I. On day 7, Lung auscultation revealed moist rates over the lungs base. Chest x-ray revealed double lung appearance with high-density scattered patch shadow in both girls. Echocardiography revealed normal coronary arteries in both of them. They were treated with cefuroxime axetil, 100mg/kg/day in two divided doses for three days. The temperature continued to remain high for next three days. A repeat echocardiography on day 10 demonstrated enlargement of the right coronary artery in the younger one (coronary artery diameter of 3.3 mm, the normal value: 1.74 mm-2.21mm) and pericardial effusion in the elder one. The diagnoses of incomplete KD^1^ and pneumonia were considered and intravenous immunoglobulin (IVIG), 1g/kg/day for 2 days was administered on day 10. Oral aspirin and dipyridamole were started subsequently. Aspirin 30 mg/kg/day was started, which was given for 2 weeks and reduced to 5 mg/kg/day and continued for 6 additional weeks. Dipyridamole 5 mg/kg/day was given for 4 weeks. The temperature returned to normal within 24 hours. On day 14 repeated echocardiography revealed enlargement of the right coronary artery in the elder one (diameter of 2.9 mm). Echocardiography on day 20 demonstrated normal diameter of right coronary arteries in both girls. Further follow ups at 3 months, 6 months and one year after onset of the disease demonstrated normal echocardiography findings.

**Table-I:** The clinical manifestations and test results of Kawasaki disease in two sets of MZ twins

*Clinical Findings*	*Twin set 1*		*Twin set 2*	
	*Younger*	*Elder*	*Younger*	*Elder*
Age at onset (days)	71	71	540	593
Sex	Female	Female	Male	Male
Fever＞5 Days	+	+	+	+
Rash	+	+	+	+
Bilateral conjunctival congestion	-	-	+	+
Peripheral Changes	+*	+*	+**	+**
Oral Changes	-	-	+^&^	+^&^
Lymphadenopathy	+	+	+	+
Coronary Artery Changes	+^#^	+^##^	-	-
IgG antibodies to the herpes simplex virus	+	+	-	-
Blood culture	negative	negative	negative	negative
Highest WBC	21.9 × 10^9^ / L	13.2×10^9 ^/ L	25.5 × 10^9^ / L	14.3 × 10^9^ / L
Erythrocyte sedimentation rate	/ hr	/ hr	/ hr	/ hr
CRP	193 mg / L	178 mg / L	157 mg / L	73.8 mg / L
Chest X-Ray	pneumonia	pneumonia	bronchitis	bronchitis

*Acute phase indurative edema and membranous desquamation from the fingertips in the convalescent phase. **Indurative edema in hands and feet. ^&^Red lips and strawberry tongue. ^#^The right coronary artery ectasia, widest diameter .

^##^The right coronary artery ectasia, widest diameter . ESR Erythrocyte sedimentation rate, CRP C-reactive protein, WBC white blood cell count, “ + ” = exists, “ – ” = does not exist.

***Twin set 2:*** The younger of 18-month-old MZ twin boys presented with fever and cough of 8 days duration and rash of three days duration. On day 8, lung auscultation revealed harsh breath sounds and chest x-ray revealed double increased pulmonary markings. After 53 days, his elder brother was also admitted to hospital with a fever and cough of 6 days duration. Chest X-ray on day 6, revealed similar features as observed in his brother. Clinical manifestations and findings of part of the laboratory tests and echocardiography are shown in Table-I. Both boys were treated with cefuroxime axetil 100mg/kg/day for three days. The temperature in both continued to remain high. The clinical manifestations met the criteria of diagnosing KD.^1^ The diagnoses of KD and bronchitis were considered. IVIG 1g/kg/day for 2 days was given to younger one on day 8 and to elder one on day 9 of admission. Oral aspirin and dipyridamole were given subsequently for both. Aspirin 30 mg/kg/day was started, which was given for two weeks and reduced to 5 mg/kg/day and continued for 4 additional weeks. Dipyridamole 5 mg/kg/day was given for two weeks. Temperature returned to normal within 12 hours. Serial echocardiography revealed normal diameter of coronary arteries in both twins. Further follow ups at three months, six months and one year after disease onset demonstrated normal echocardiographic findings.

## Discussion

 KD is rare in children younger than 6 months.^5^ Incidence is highest in Japan, even if Japanese migrate to areas with lower incidence, morbidity remains high. Such as in the incidence of KD in children younger than 5 years in Japanese Americans in Hawaii, of 135/100 million is equivalent to the incidence reported in Japan.^6^ KD has a tendency for familial aggregation. The prevalence of a recurrence of KD and the incidence rates involving siblings of patients whose parents have a history of the disease are higher than those in the general population of children.^7^ The two sets of MZ twins in the present study were selected from 577 KD patients over 10 years in Jilin Province, China.^8^ Kottek A opined that susceptibility to KD is influenced by host genetics.^4^

 In MZ twins who are discordant for clinical signs of KD, sub-clinical coronary artery vasculitis may be present. Health care providers should therefore consider laboratory testing and echocardiography in both MZ twins when one twin presents with clinical KD.^4^ The younger one of twins in both families showed relatively more severe clinical symptoms, which suggests that, although the genetic material of MZ twins is similar, subtle differences in the genetic material between individuals might play a role in the pathogenesis of the disease. Compared with MZ twins of Twin set 2, those of Twin set 1 were found to have right-coronary-artery ectasia, a situation suggesting that genes susceptible to coronary-artery lesions may exist in Twin set 1.^9^ The results regarding the susceptibility of genes for KD and coronary artery lesions in these two families are not yet available.

 Meanwhile, the clinical and epidemiological features of KD strongly suggest an infectious cause, and the laboratory results support it.^10^ Despite scholars carrying out studies on various infectious factors of KD over the last 40 years, consistent and satisfactory results have not been obtained. This suggest that the pathogen that causes KD is a microorganism omnipresent in the natural environment, that older children and adults have acquired immunity after infection by the unknown pathogens(s) and that most individuals infected are asymptomatic. In infants and younger children the acquired immunity may not be adequate and KD may be induced by some pathogens as seen in the two sets of twins. In the two sets of twins, although microorganisms had not been detected (blood cultures were negative), the clinical features strongly imply respiratory tract infection. A simultaneous onset and identical clinical manifestations in Twin set 1 suggest an exposure to same pathogenic factors. Identical clinical features, but of a sequential onset and older age group in Twin set 2 suggesting an impact of environmental differences. MZ twins provide the best samples for studying the impact of environmental factors on the disease because the role of genetic factors can be partially ruled out between MZ twins.

 There was pulmonary infection in two sets of MZ twins, suggesting that the respiratory tract may be one of the channels by which the pathogen(s) enter the body and cause KD.^11^ It is also likely that an infection triggers KD in individuals with genetic susceptibility.

 MZ twins in Twin set 1 tested positive for IgG antibodies to the herpes simplex virus but we did not detect any 4-fold rise in double serum IgG, and IgM antibodies. As infants at age of 71 days may show IgG antibodies transferred from the mother, it is not possible to infer that the herpes-simplex-virus infection caused the KD.

 In conclusion, our observation supports the possibility that existence of genes susceptible to KD and coronary-artery lesions and diverse and complex nature of pathogenesis of coronary artery lesions in KD. The onset of KD after infection and the incidence of coronary artery lesions is discordant between the individuals of MZ twins.
